# Theta waves in children’s waking electroencephalogram resemble local aspects of sleep during wakefulness

**DOI:** 10.1038/s41598-017-11577-3

**Published:** 2017-09-11

**Authors:** Sara Fattinger, Salome Kurth, Maya Ringli, Oskar G. Jenni, Reto Huber

**Affiliations:** 1 Child Development Center, University Children’s Hospital Zurich, Zurich, Switzerland; 2 Children’s Research Center, University Children’s Hospital Zurich, Zurich, Switzerland; 3 Neuroscience Center Zurich, Zurich, Switzerland; 4 Pulmonary Clinic, Division of Pulmonology, University Hospital Zurich, Zurich, Switzerland; 5 Department of Child and Adolescent Psychiatry and Psychotherapy, Psychiatric Hospital, University of Zurich, Zurich, Switzerland

## Abstract

Vyazovskiy and colleagues found in rats’ multi-unit recordings brief periods of silence (off-states) in local populations of cortical neurons during wakefulness which closely resembled the characteristic off-states during sleep. These off-states became more global and frequent with increasing sleep pressure and were associated with the well-known increase of theta activity under sleep deprivation in the surface EEG. Moreover, the occurrence of such off-states was related to impaired performance. While these animal experiments were based on intracranial recordings, we aimed to explore whether the human surface EEG may also provide evidence for such a local sleep-like intrusion during wakefulness. Thus, we analysed high-density wake EEG recordings during an auditory attention task in the morning and evening in 12 children. We found that, theta waves became more widespread in the evening and the occurrence of widespread theta waves was associated with slower reaction times in the attention task. These results indicate that widespread theta events measured on the scalp might be markers of local sleep in humans. Moreover, such markers of local sleep, seem to be related to the well described performance decline under high sleep pressure.

## Introduction

Sleep and wakefulness are clearly separable brain states, but are yet critically dependent on each other. The separation of the two states has not only been defined on the behavioural, but also on the electrophysiological level. Physiological deep sleep is associated with a slow oscillating pattern of brain activity and the absence of physical activity, while waking behaviour is associated with continuous brain activity^1^. The dependence on each other is best illustrated by the homeostatic regulation of sleep found in animals and humans. Sleep need grows with time spent awake and can only be dissipated during sleep^2^. For both aspects, the build-up of sleep need and its dissipation during sleep, established electrophysiological markers exist. The build-up of sleep need is reflected in electroencephalographic (EEG) theta activity (EEG power between 6–8 Hz) during wakefulness^3–6^. Slow wave activity (SWA ﻿1–4.5 Hz) during sleep, on the other hand, reflects the level of sleep need at the beginning of the night and its consecutive recovery across the night^7^. A key difference between the two vigilance states deep sleep and wakefulness is the change in properties of neurons. Compared to the tonically active state of wakefulness, the membrane potential of thalamo-cortical neurons become bistable (i.e. alternating between two different voltage level) during deep sleep, oscillating between depolarization (on-state) and hyperpolarization (off-state)^1^. As a result, neurons display an alternating activity pattern of periods (100–500 ms) with neuronal activity and periods of complete neuronal silence (100–300 ms)^8^.

However, most recent findings have challenged the concept of dichotomy of sleep and wakefulness on the neuronal level. Evidence for wakefulness-like brain activity during sleep and local sleep during wakefulness has been presented in human and animal data^9–11^. For example, intracranial recordings of brain activity in patients suffering from drug resistant epilepsy show clear signs of wakefulness-like activity typically observed over sensorimotor areas^9^. Using multi-unit recordings in rats, Vyazovskiy *et al*.^10^ described brief intermittent periods of silence (off-states) in local populations of cortical neurons during wakefulness. In contrast to the globally occurring slow oscillation pattern across almost all cortical neurons during deep sleep, the off-states during wakefulness seem to occur rather locally and last only 50–100 ms. Vyazovskiy *et al*.^10^ also explored the characteristics of these brief periods of silence during prolonged periods of wakefulness and found that with increasing sleep need the local off-states became more frequent and involved larger cortical areas (i.e. became more widespread) which was associated with increased theta activity in the surface EEG. Interestingly, in the human waking EEG, the increase in theta activity after sleep deprivation correlated with impaired performance^12–14^. Hence, the question arises whether local sleep (off-states) of cortical neurons during wakefulness, which seem to reflect waking theta activity, can account for the performance impairment. To address this question, Vyazovskiy *et al*.^10^ trained rats on a sugar pellet reaching task. The results showed significantly more off-states (300–800 ms) prior to an unsuccessful reaching attempt as compared to successful trials. These data indicate that local populations of cortical neurons that “fall asleep” may be responsible for the impaired performance following sleep deprivation.

All of the above mentioned findings have been drawn from intracranial recordings close to the neuron. In this study, we investigated whether the surface EEG likewise provides evidence for such local intrusion of one brain state into the other that is local sleep during wakefulness. Thus, we analysed high-density (hd, 128 channels) wake EEG recordings which provide a good spatial resolution and examined children because they generally show a pronounced increase in sleep need and a high signal to noise ratio.

## Results

### Pronounced increase of theta activity from morning to evening in children

**Figure 1:**
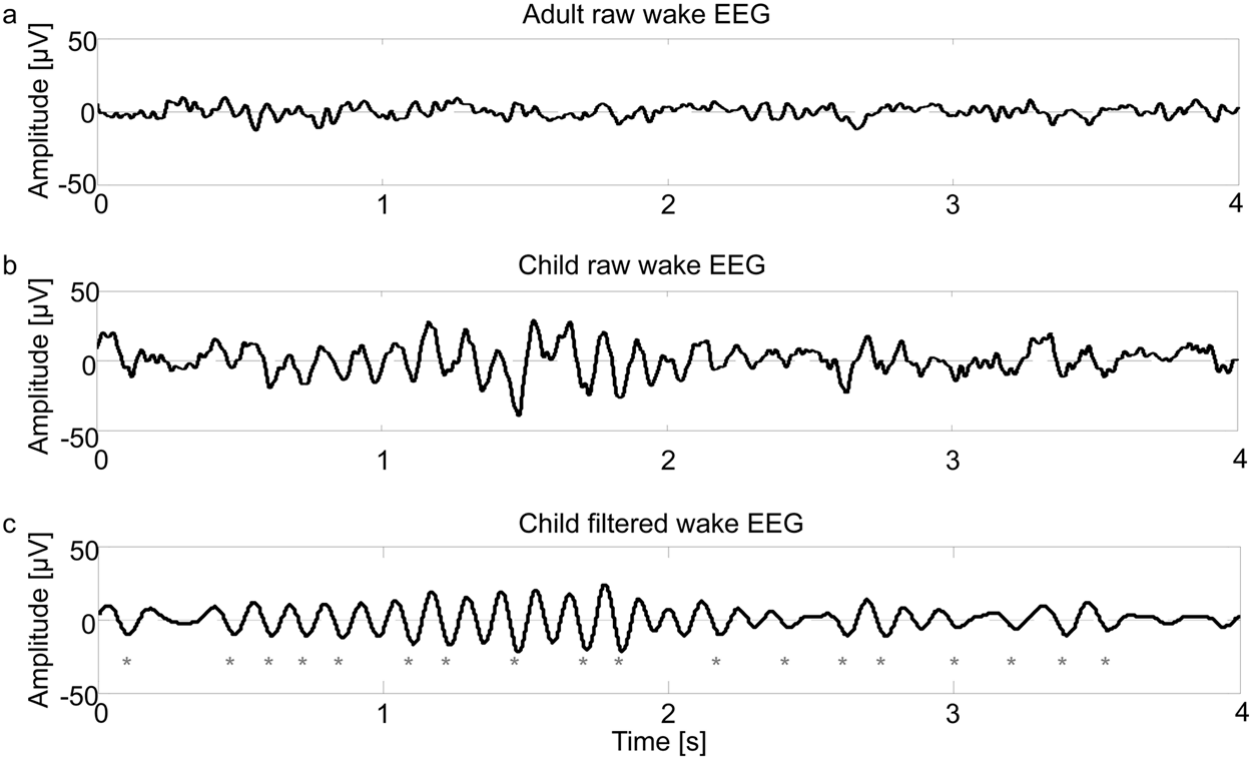
Representative examples of amplitude differences in wake EEG of a child and an adult. (**a**) Four seconds of the unfiltered wake EEG of an adult in the evening (male subject, 19.4 years old, from the data set presented in ref. 45). (**b**) Four seconds of the unfiltered wake EEG of a child in the evening (male subject, 12.6 years old). Note, the amplitude of the wake EEG is much higher in children compared to adults. (**c**) Example of the theta event detection algorithm: the same 4 seconds EEG as in b are displayed after filtering (Chebyshev Type 2 Filter: pass-band 5 and 9 Hz, stopband: below 4 and above 12 Hz). Grey asterisks mark the theta events detected by the algorithm.

We report data from 12 healthy right-handed children (3 females, 10.5 ± 0.3 years of age, age range from 8.8 to 12.6 years). Hd-EEG were recorded during a 4-min auditory attention task in the morning and evening. Independent of the time of day (i.e. morning or evening), the amplitude of the wake EEG is much higher in children compared to adults (for an illustrative example see Fig. 1). Thus children’s EEG provides a much better signal to noise ratio, which facilitates the reliable detection of single theta events in the wake EEG. Moreover we observed a pronounced increase of theta activity from morning to evening by 44.8 ± 5.6% in our participants, indicating that sleep need is accumulating fast in children. Note, this power increase in the evening compared to the morning was not specific for the theta-band. Spectral analysis (0.25–25 Hz) revealed increased power values for almost the entire frequency range (see Fig. S3). Since the purpose of the current study was to specially quantify the property of theta events in the waking EEG as a sign of local sleep (i.e. underlying short local neuronal off-states) further analysis was focusing on the theta-band only. In the following steps, we established a method characterizing theta events in the waking EEG.

### Theta event detection and cluster size of theta events in the morning and evening

**Figure 2:**
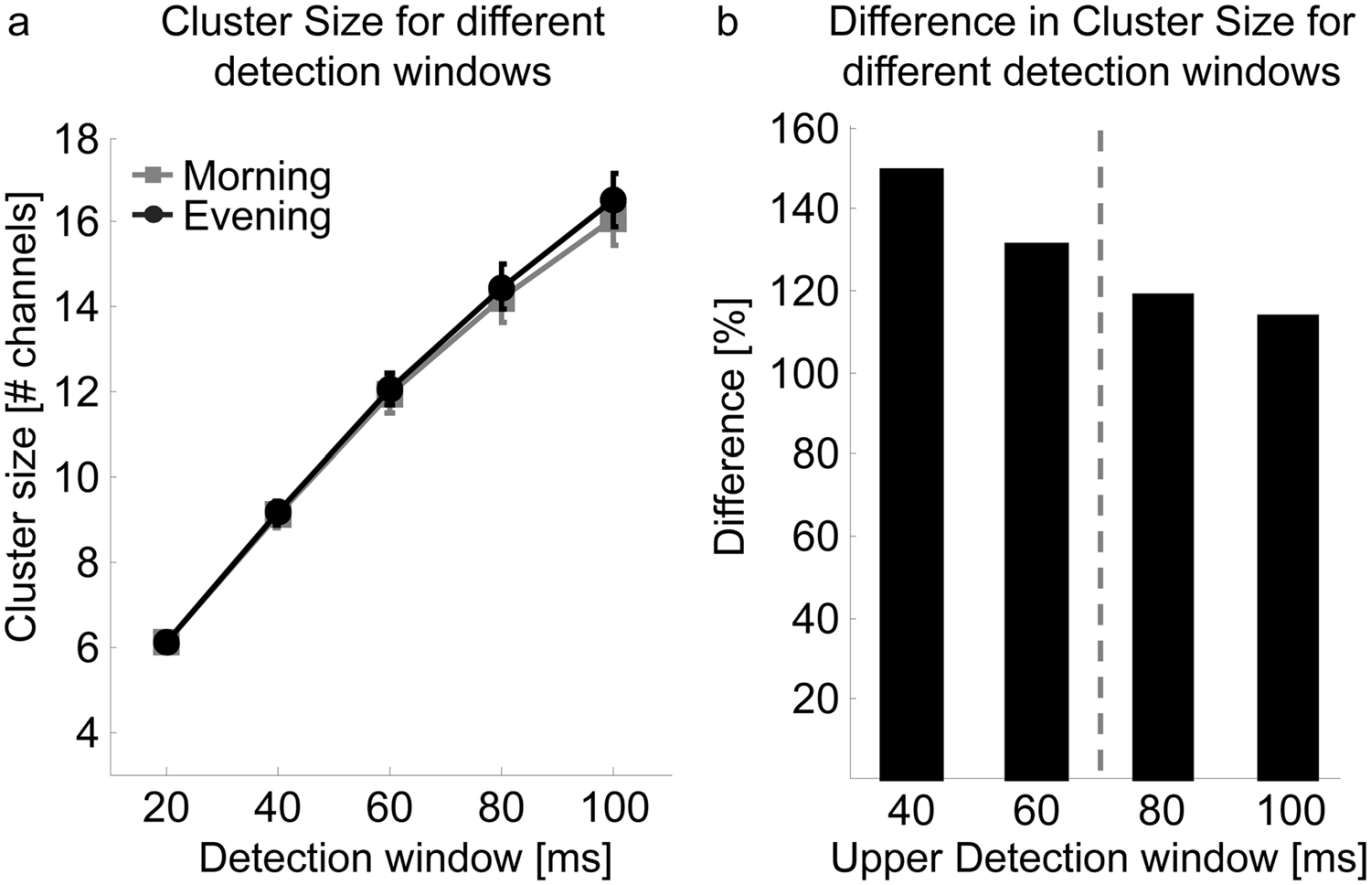
Relationship between detection window size and cluster size. (**a**) Mean cluster size of theta events for different detection windows, based on an amplitude detection threshold matched to each EEG recording (mean ± SEM of cluster sizes for each detection window in the morning and evening are shown). The larger the detection window, the more channels are involved in the cluster sizes (linear mixed effect model: F_detection window_ = 546.92, p < 0.001, n = 12). No difference in cluster size of theta events was found between morning and evening (linear mixed effect model: F_time_ = 0.01, p = 0.93 n = 12). (**b**) The difference (percentage) of mean cluster sizes (i.e. the number of channels involved in a cluster) between different detection windows. Morning and evening are pooled. X-axis represents upper detection window (i.e. 40 = cluster size_Detection window 40_/cluster size _Detection window 20_*100). A detection window of 60ms (indicated by the grey dotted line) was selected for analysis.

In a first step, we investigated whether theta events in the wake EEG are more global in the evening compared to the morning. Thus, single theta events and their cluster sizes (i.e. number of channels involved in a theta event) were detected (for details see Methods). Similarly to the travelling of slow waves across the cortex^15^, we assumed that theta events are initiated at any site and spread across the cortex. As expected, a close relationship was found between cluster size and length of the detection window: the longer the detection window the larger the cluster size of theta events (F_detection window_ = 546.9, p < 0.001, n = 12, Fig. 2a). Yet, independent of the considered detection window, the cluster size of theta events was similar in the morning and evening (F_time_ = 0.01 p = 0.93; n = 12, Fig. 2a). Moreover a similar number of theta events were detected in the morning and evening (mean across all detection windows evening: 2499 ± 137.75; morning: 2549 ± 143.5, p = 0.75, n = 12). The similarity in these parameters between morning and evening is likely a result of matching the amplitude detection threshold to each EEG recording (i.e. in the morning a lower amplitude threshold was needed to detect theta events compared to the evening: detection threshold morning: 3.34 ± 0.17 µV; evening: 3.92 ± 0.26 µV; p < 0.001, n = 12, for further details see Methods). Because no differences in cluster sizes were found between morning and evening, all cluster sizes for each detection window were pooled across time points. Differences in the number of channels involved in a cluster across the different detection windows were computed (Fig. 2b). As shown in Fig. 2b, the increased number of channels involved in a cluster with increasing detection windows seems to level off after 60 ms. Thus, for further analysis we focused on a detection window of 60 ms. To exclude the possibility of a selection bias related to the detection window, we performed the same analysis for a detection window up to 100 ms (see supplementary information).

### The cortical involvement of theta events increases from morning to evening

**Figure 3:**
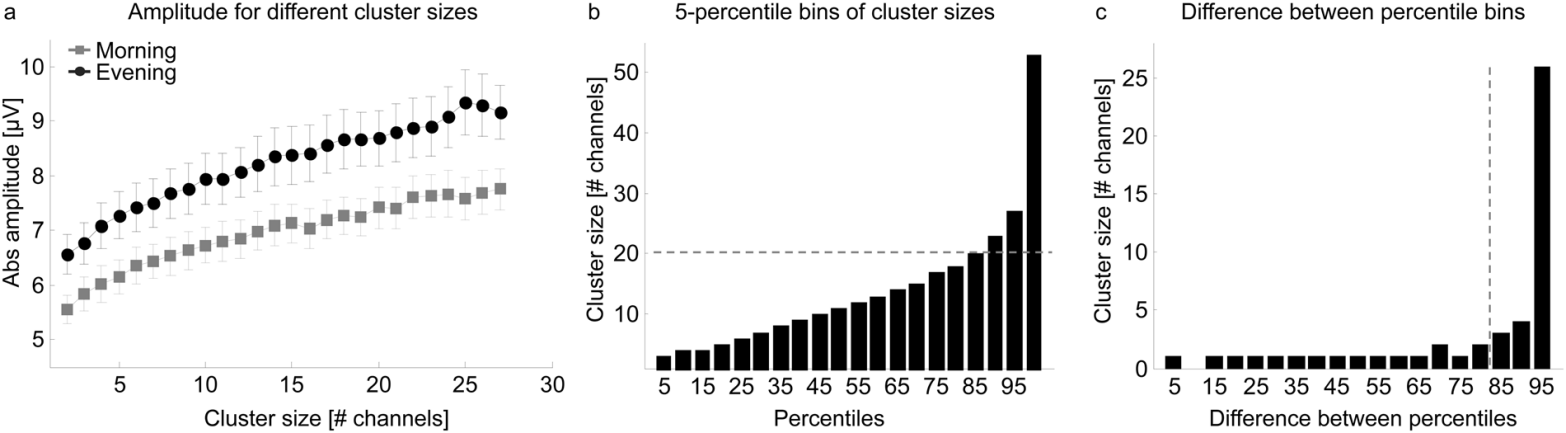
Definition of widespread theta events based on a detection window of 60 ms. (**a**) Relationship between amplitude and cluster size in the morning and evening (mean ± SEM of the amplitude for each cluster size are presented for morning and evening. For each subject, mean amplitude was calculated when at least 5 theta events for the given cluster size were detected). Larger amplitudes were associated with increased cluster sizes (linear mixed effect model F_cluster size_ = 32.61, p < 0.001, n = 12). For any cluster size a larger amplitude was detected in the evening compared to morning (linear mixed effect model: F_time_ = 207.01, p < 0.001, n = 12). (**b**) 5-percentile bins of cluster sizes (morning and evening pooled). The 85^th^ percentile corresponds to a cluster size of 20 channels (grey dotted line). (**c**) Difference in the number of channels involved in a cluster size between each 5-percentile bin from Figure **b**. Numbers on the x-axis indicate the lower bin (i.e. 5 corresponds to the difference between the 5^th^ and the 10^th^ bin). Because the number of channels involved in the cluster size are increasing from the 85th percentile, the 85th percentile (corresponding to a cluster size of 20 channels, see **b**) was used for cluster size cutoff definition for widespread theta events.

Next, we elaborated the relationship between cluster size and theta wave amplitude. Independent of the time of day, a higher amplitude was associated with a larger cluster size (F_cluster Size_ = 32.61, p < 0.001, n = 12; detection window 60 ms; Fig. 3a). For any cluster size, a higher amplitude was generated in the evening compared to the morning (F_time_ = 207.1, p < 0.001, n = 12; Fig. 3a; similar results were obtained with a detection window of 100 ms, Fig. S1). This result indicates that in the evening smaller clusters are needed to generate the same theta wave amplitude as compared to the morning. In a next step, we aimed at further investigating the cortical involvement of theta waves at the two time points by calculating the number of widespread theta events. Our data analysis showed that for a definition of a “widespread theta event” the cluster size as well as the amplitude of the wave have to be taken into account. Thus, to provide an unbiased view on the definition of a cluster of electrodes involved in a widespread event we performed two specific analyses.

#### Definition of cluster size threshold for widespread theta events

We pooled data across the two time points and divided all cluster sizes into 5-percentile bins (Fig. 3b). Next, the difference in the number of channels involved in a cluster between each 5-percentile bin was computed (Fig. 3c). For each increasing 5-percentile step, the cluster consisted of one or maximally two additional channels. However, exceeding the 85^th^ percentile, the number of channels involved in a cluster increased stronger. We therefore defined the cluster size at the 85^th^ percentile as the cutoff defining widespread theta events. This cutoff corresponds to a cluster of 20 channels. In other words, a minimum of 20 channels involved in a theta event accounted for a “widespread theta event”.

#### Definition of amplitude threshold for widespread theta events

When defining an amplitude threshold for widespread theta events, it needs to be considered that a cluster of 20 channels generates a smaller amplitude theta wave in the morning compared to the evening (see above). Thus, to ensure that the definition of widespread theta events is not biased towards the evening, this amplitude difference was taken into account. The mean amplitude, which was generated by a cluster size of 20 channels, was calculated for the morning session in each subject and this value was considered as amplitude threshold for the morning and evening sessions.

### Widespread theta events in the morning and evening

**Figure 4:**
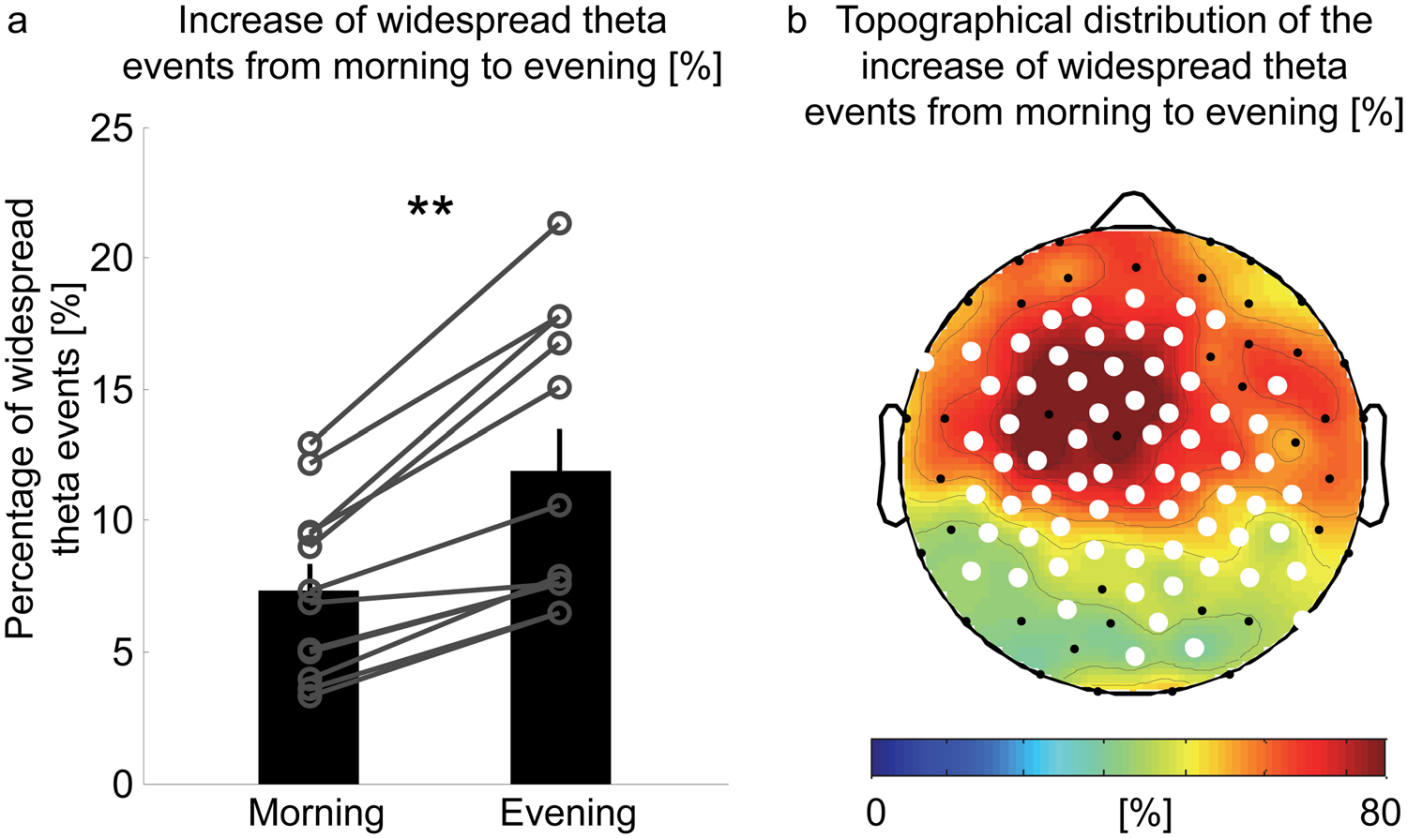
Increase of widespread theta events form morning to evening. (**a**) Percentage of widespread theta events in the morning and evening (bars represent mean ± SEM, circles represent individual subjects). Percentage of widespread theta events increase in all subject from morning to evening (p < 0.001, paired Student’s T-Test, n = 12). (**b**) Topographical distribution of widespread theta events﻿ ﻿changes from morning to evening (evening-morning/morning*100). Percentage of widespread theta events increases globally from morning to evening, which is most pronounced over central and frontal areas (white dotes p < 0.05, paired Student’s T-Test, n = 12, after nonparametric cluster-based statistical testing).

Next, we applied both thresholds (amplitude and cluster size) to morning and evening sessions. The results show that the mean cluster size of widespread theta events did not differ between morning and evening (morning: 26.0 ± 0.2 channels, evening: 25.9 ± 0.2 channels, p = 0.2, n = 12). Moreover, in the morning as well as in the evening, only a minority (~10%) of all theta events were “widespread” (Fig. 4). We finally calculated the percentage of widespread theta events for both time points and found a significant increase from morning to evening. Widespread theta events increased in each subject by an average of 64.3 ± 7.1% (p < 0.001, n = 12, Fig. 4a). Moreover, this increase appeared “globally”, as detections occurred in a large proportion of channels (Fig. 4b), and was most pronounced across central and frontal areas. Similar results were obtained when considering a detection window of 100 ms (percentage of widespread theta events morning: 9.7 ± 1.0%, evening: 16.7 ± 1.7%, p < 0.001, n = 12, with a cluster size threshold of 24 channels, see Fig. S1).

### Widespread theta events are associated with slower reaction times

Then we aimed at exploring whether widespread theta events are associated with impaired performance. Reaction times were similar between morning and evening (morning: 320.7 ± 25.9 ms; evening: 323.6 ± 19.3 ms, Wilcoxon rank sum test: p = 0.8286, n_morning_ = 8; n_evening_ = 10; Wilcoxon signed rank test: p = 0.69, n = 6, for reason of subject exclusion see Methods). Therefore, for this analysis, the data from morning and evening were pooled, since we wanted to investigate whether the occurrence of global theta events per se (i.e. sign of local sleep independent of time of day) might underlie performance impairments.

**Figure 5:**
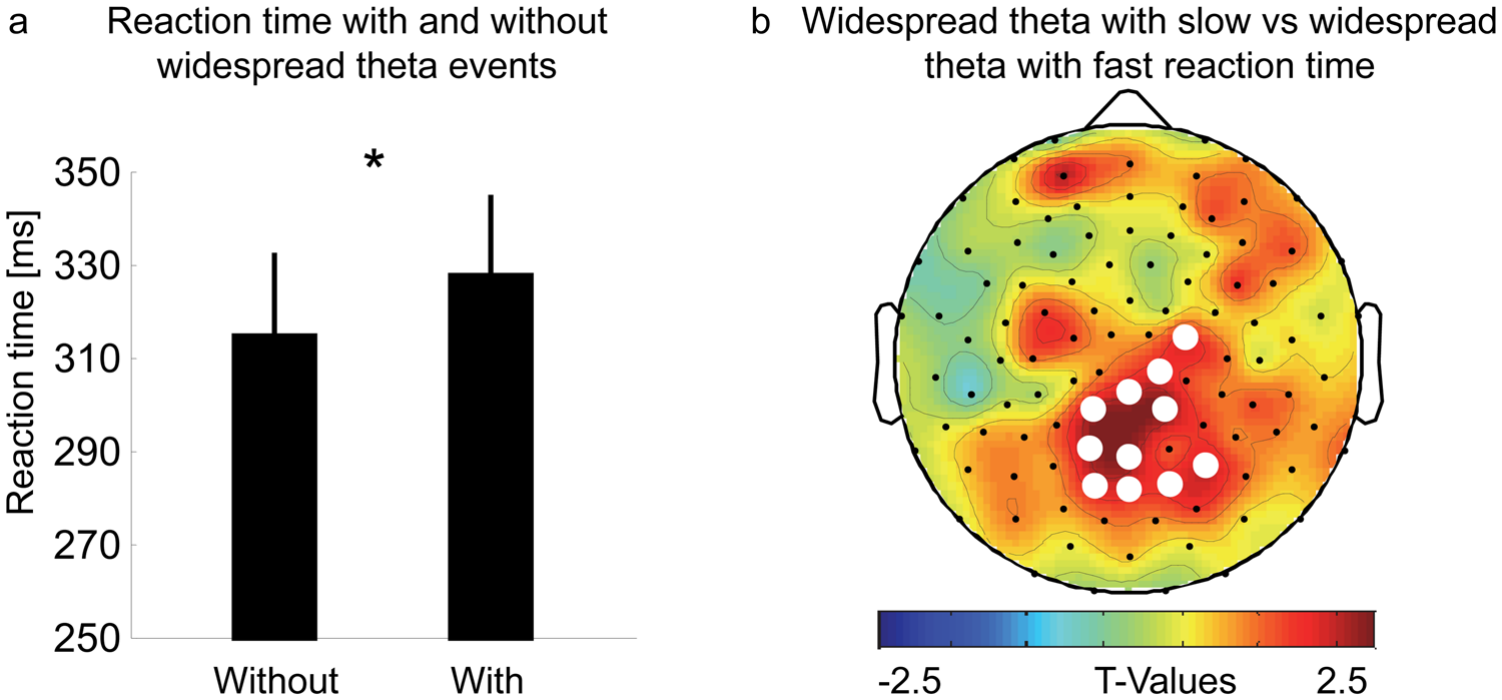
Relationship between widespread theta events and reaction time. Data from morning and evening pooled. (**a**) Mean reaction time with no widespread theta event (=Without) and with at least one widespread theta event (=With). Reaction times associated with widespread theta are slower compared to reaction times without widespread theta events (mean ± SEM, p = 0.036, paired Student’s T-Test, n = 18). (**b**) Topographical comparison of widespread theta events and reaction time. All reaction times were split into two groups: the fast reaction time (defined as the fastest 40% reaction times) and the slow reaction time group (the slowest 40% reaction times, including the missed stimuli). For each group it was evaluated how often a channel was involved on average in a widespread theta event. A cluster of 11 channels (white dotes) was found, which were significantly more involved in widespread theta events in the slow reaction time compared to the fast reaction time group (white dotes p < 0.05, paired Student’s T-Test, n = 18, after nonparametric cluster-based statistical testing).

Thus, theta events were detected within a time window starting 900 ms prior and ending 100 ms after the deviant tone. Within each subject all reaction times were grouped according to the following criteria: all reaction times associated with at least one widespread theta event were defined as “reaction times with widespread theta” and all reaction times in which no widespread theta event were detected were defined as “reaction time without widespread theta”. The number of reactions did not differ between the two groups (number without widespread theta: 11.0 ± 1.0; with widespread theta: 12.3 ± 1.0, p = 0.52). However, reaction times associated with widespread theta event were on average 12.88 ± 5.49 ms slower compared to reaction times without widespread theta events (p = 0.036, n = 18; Fig. 5a). Note, this finding was not biased by a few individuals which were included twice due to the pooling of morning and evening (see Fig. S2 for individual data).

### Regional association between widespread theta events and impaired performance

Finally, we investigated whether this association between widespread theta events and slower reaction times was related to specific topographical areas. Theta events were grouped into fast reaction time (defined as the fastest 40% of reaction times) and slow reaction time groups (the slowest 40% reaction times, including the missed trials). For each group, the frequency of channels involved in a widespread theta event was calculated. We found a cluster of 11 channels located over premotor cortex (Brodmann area 6, 5 channels), parietal cortex (Brodmann area 7, 4 channels) and somatosensory cortex (Brondmann area 5 & 3; 2 channels), which were more frequently involved in a widespread theta event in the slow reaction time compared to the fast reaction time group (slow reaction time group, 0.49 ± 0.08; fast reaction time group, 0.32 ± 0.07; p = 0.02; Fig. 5b).

## Discussion

We report a ~45% increase of waking theta activity in the evening compared to the morning. In order to explore the underlying mechanisms of these changes in theta activity we ran single theta events detection and compared theta properties between morning and evening. Results show that widespread theta events are more likely occurring in the evening compared to the morning. Furthermore, widespread theta events were associated with slower reaction times in a simple auditory attention task. Thus, single theta events recorded in the human scalp EEG during wakefulness show properties comparable to the local off-states in multi-unit recordings of rats^10^. We propose that widespread theta events represent markers for local sleep during wakefulness.

The gold standard electrophysiological marker of homeostatic sleep regulation is SWA during NREM sleep, which increases as a function of prior wakefulness^7^. However, also the waking EEG informs about the homeostatic regulation of sleep: Several studies in adults revealed a pronounced increase of theta activity in the course of sleep deprivation^3, 4, 6, 14, 16^. Moreover, the increase in theta activity was related to the increase of SWA during initial recovery sleep^3–6^. Furthermore, theta activity parallels subjective sleepiness and thus represents an objective measure of sleep need during wakefulness^17–21^. Notably, we also observed a ~45% increase of theta activity in the evening compared to the morning in our children. In contrast, theta activity in adults is only marginally increased in the evening compared to the morning^5, 22^. This might be due to a faster build-up of homeostatic sleep pressure during the day in children^23–25^.

Investigating neuronal activity by multiunit activity in rats uncovered an interesting feature of scalp theta activity^10^. During wakefulness, cortical neurons are tonically depolarized and fire irregularly giving rise to an EEG pattern displaying low-amplitudes and fast-activities^26^. In contrast, during NREM sleep virtually all cortical neurons are oscillating between an active on- and a silent off-state^1^ leading to the prominent slow waves in the EEG^26^. When carefully aligning EEG theta waves during wakefulness with neuronal activity, subsets of cortical neurons showed brief intermitted periods of neuronal silence, even though the global EEG and behaviour show typical waking activity. These off periods were essentially indistinguishable from the off-states underling sleep slow waves. Thus, theta waves measured on the surface seem to be associated with the occurrence of local, sleep like off-states on the neuronal level. Moreover, with increasing sleep pressure during sleep deprivation, these off-state became more frequent and more global, involving different subsets of cortical neurons spread over the cortex^10^. Also in our sample the majority of theta events (~90%) occurred in locally restricted areas (8.7 ± 0.2 channels, morning and evening). Nevertheless, in every child, the percentage of widespread theta events was increased in the evening compared to the morning, indicating that the likelihood of widespread theta events increased with time spent awake. Moreover, this increase of widespread theta events from morning to evening was most pronounced over central and frontal areas which is in line with previous studies observing similar regional changes in theta activity due to sleep deprivation in adult subjects^14, 27^.

Performance impairments due to increased sleep need have been well described^14, 28–30^. Unquestionably, micro sleep, the intrusion of brief periods of electrophysiologically classified sleep into wakefulness results in behavioural deterioration with potentially dramatic consequences, for instance when driving^16, 31^. Thus, a key question is whether short local off-states during wakefulness are also associated with behavioural consequences. Indeed, Vyazovskiy *et al*.^10^ have shown that off-states during wakefulness result in impaired performance. When rats were performing a sugar pellet reaching task, the number of missed trials increased under high sleep need condition. Most importantly, missed trials were associated with local off periods in the primary motor cortex^10^. Our findings are in line with these observations. In a preliminary analysis, we could show that preceding occurrence of more widespread theta events is related to a worse performance. More specifically, reaction times associated with widespread theta were longer compared to reaction times without widespread theta. Moreover, this relationship between reaction time and widespread theta was most pronounced over brain regions associated with motor execution (Fig. 5b). Because the oddball paradigm is primarily targeting the auditory system, it might be surprising that no differences over auditory cortex was observed. One possible explanation for this observation might be that the occurrence of widespread theta events was directly compared between fast and slow reaction times. Thus, the auditory stimuli were the same for both conditions, whereas motor executions (i.e. slow vs. fast reaction times) were different. Moreover, Gorgoni and colleagues^14^, also observed a frequency specific correlation between increased theta activity and impaired performance in the psychomotor vigilance task (PVT) over centro-posterior brain areas after sleep deprivation in adults. As in our task, in the PVT, subjects have to react with a mouse click (i.e. motor execution) to a visual stimulus.

Notably, overall reaction times were within a normal range (mean reaction time with widespread theta: 328.1 ± 16.1ms; without widespread theta: 315.2 ± 16.5ms). In addition, overall only 10 missed clicks (5 in the evening and 5 in the morning) were detected, which makes the analysis of behavioral omission (i.e. similar to the study of Vyazovskiy and colleagues^10^) due to widespread theta events difficult. Alternative tasks, like the task-switching paradigm, which is highly susceptible to sleep restriction^32^, may be more sensitive to assess worsening of performance due to the occurrence of local sleep. These initial findings, however, supported the suggestion that the occurrence of local sleep, measured as widespread theta events on the scalp surface of the awake brain, seem to be related to some cognitive impairments.

How neurons respond to incoming stimuli may depend on whether neurons are in an on- or off-period, which in turn may affect cortical functioning^33, 34^. Strikingly, overall performance in sleep deprived subjects is characterized by fluctuating performance levels (i.e. alternating between normal and very bad performance)^29, 35^. The stochastic, all-or-none occurrence of off-periods in cortical neurons could indeed account for such fluctuations. Thus, the well-known negative consequences on performance when staying awake too long^30, 36^ may relate to the occurrence of local off-states during wakefulness. The correlation between increased theta activity in the waking EEG and performance decrease in different studies may support this conclusion^12, 13^. Unfortunately, our data does not allow to establish a direct relationship between theta activity and performance changes because of too short inter-stimulus intervals.

The last part discusses possible mechanisms underlying the increase in local sleep under conditions of high sleep need. Computer simulations of sleep slow waves show that amplitude and slope of slow waves are directly related to the number of neurons involved in on- and off-states. The more neurons enter an on- or off-state near synchronously, the higher the amplitude^37, 38^. This is in line with our observation that higher amplitude theta waves are associated with increased cluster size, independent of the time of the day. If theta waves in the wake EEG underlie similar mechanism as slow waves during sleep, this result might indicate that higher amplitude theta waves might be generated by a larger subpopulation of cortical neurons simultaneously entering an off-state. Interestingly, theta events of the same cluster size (i.e. number of channels involved in a theta event) generated higher amplitudes in the evening compared to the morning. Computer models imply that synchrony within a neuronal network is determined by the number, strength and distribution of synaptic connections^37, 38^. Thus, the larger amplitude in the evening compared to the morning may reflect increased synaptic strength. In line with the synaptic homeostasis hypothesis^39^, higher synaptic strength in the evening facilitates synchronized neuronal activity which in turn results in higher amplitudes. Further support for this notion comes from the observation that spectral power was increased in general in the evening compared to the morning in our children (Fig. S3). Nonetheless, beside short off-states within local cortical populations, also neuronal activity of subcortical structures have been related to theta activity in the local field potential. Specifically, prominent hippocampal theta oscillations have been associated with information processing in rodents^40^. However, compared to animal studies, surface EEG recordings in humans are rather unlikely to detect subcortical activity^41^. Alternatively, a decrease in arousal-promoting neuromodulators after falling asleep may facilitate a bistable state of the membrane potential as being the case when falling asleep^42^. To what extent changes in the milieu of neuromodulators contribute to the off-stages during wakefulness needs to be further investigated. The fact that theta events during wakefulness were rather local (i.e. also widespread theta events involved only ~26 channels), might indicate that the globally active neuromodulatory system during wakefulness prevents the emergence of global bistability. Moreover, only recently it has been shown that local neuronal off-states not only occur under high sleep pressure, but also during stereotypic task performances, possibly reflecting a functional disconnection of cortical areas as a result of their disengagement^43^. However, in our dataset the number of widespread theta events was not increased in the last minute compared to the first minute of the auditory oddball task. Thus, the task might have been challenging enough for our children to prevent functional disconnection of cortical areas. The fact that overall reaction times were within a normal range and only very few missed clicks were observed further supports sustained attention of our children during the entire task.

Due to the retrospective study design, there are some limitations of the current analysis. First, the sample size of some sub analysis (i.e. performance measure) is rather low (n = 8 in the morning, n = 10 in the evening). Therefore, the interpretation that the occurrence of widespread theta events might be associated with slower reaction times remains preliminary. Nevertheless, our results indicate that widespread theta events per se (i.e. sign of local sleep) independent of time of day (morning and evening pooled, n = 18) may underlie performance impairments. Moreover, the question remains whether auditory stimuli per se (i.e. auditory processing of standard tones) have an effect on widespread theta events. Unfortunately, the inter-stimuli interval in the current study was too short for such an analysis (i.e. only 400ms before and after standard tones respectively).

In conclusion, assuming that short off-states of cortical neurons give rise to theta activity in the waking EEG, our findings suggest that in the evening, when the need for sleep is high, large subsets of cortical neurons become briefly bistable, and appear as widespread theta events in the waking EEG. According to the synaptic homeostasis hypothesis^39^ increasing synaptic strength due to learning processes during wakefulness, has various costs at the cellular and system level, such as higher energy consumption, increased cellular stress, and associated changes on supporting cells (i.e. glial cells). This accumulating costs during wakefulness seems to drive single neurons towards off-states, allowing restoring cellular maintenance^44^. However, it remains speculative whether local off-states during wakefulness in humans fulfil restorative functions.

## Methods

### Subjects and study design

The wake EEG of a subset of previously published sleep data^45^, studied at the University Children’s Hospital Zurich, Switzerland between 2008 and 2009 were analysed. All children between 8 and 13 years participating in the former study were selected. EEG was recorded during wakefulness before (evening) and after sleep (morning) during an attention task (for details see below) in 16 children. Four, out of these 16 children were excluded from the analysis because of EEG artefacts, resulting in a total number of 12 participants included for analysis. Exclusion criteria for all subjects were personal or family history of psychopathology, severe brain injury, sleep disorders, chronic diseases, and current use of psychoactive agents or other medications. Participants were not allowed to travel across more than 1 time zone in the 4 months before the study (for further details see Kurth *et al*.^45^). Written informed consent was obtained from all children and their parents, after detailed explanation of the study design and aim. The study protocol was approved by the local ethics committee (*Kantonale Ethikkommission Zürich, Switzerland, StV 27/07*) and was performed according to the Declaration of Helsinki.

### Wake EEG Recording

Hd wake EEG (Electrical Geodesics Sensor Net for long-term monitoring, HydroGel, 128 channels, Electrical Geodesics, Eugene, OR, USA) was recorded for 4 minutes during an attention task in the evening right before going to bed and in the morning ~30 minutes after wake up. To ensure habitual levels of sleep need in the evening, subjects kept a regular sleep-wake cycle 7 days prior to the recording. Compliance with the schedule was verified by daily sleep diaries completed by the subjects or parents and the recording of activity by means of wrist actigraphy. No naps were allowed 24 hours before the recording day. Sleep was scheduled individually according to subject’s reported habitual bed times. In the morning subjects were woken up in order to attend school. For the EEG recording the electrode net was adjusted to the vertex and the mastoids, and all electrodes were filled with electrolyte gel. Impedances were measured prior to EEG recordings in the evening and morning and kept below 50 kΩ.

### Attention task

During the wake recording subjects performed an attention task based on an auditory oddball paradigm. Subjects were instructed to sit quietly in front of a screen and fix a white cross on a black background. Over a period of 4 min, participants listened to 300 stimuli (~80 dB) with an inter-stimuli interval of 0.8 s, whereof 90% were standard tones (880 Hz) and 10% were deviant tones (988 Hz, presented in random order). Subjects were asked to respond to the deviant tones with a mouse click as quickly as possible. The task was programed with MATLAB (Math Works, psych toolbox). As performance measure, the reaction time to deviant tones was assessed. For performance data, four subjects in the morning and 2 subjects in the evening were excluded due to technical issues related to the simultaneous acquisition of behavioural and EEG data. Thus, data of 6 subjects was available for both, the morning and evening.

### Pre-processing of the wake EEG recording

EEG data were sampled at 1000 Hz (0.01–400 Hz), and recorded to the vertex (Cz). For offline analysis, the EEG signal was pre-processed similar to Hung *et al*.^6^ After passband filtering (0.1–48 Hz), poor-quality channels were identified by an automatic outlier detection based on amplitude threshold criteria, and were confirmed by visual inspection. Rejected channels were interpolated using spherical splines (NetStation, Electrical Geodesic Inc.). Next, data were transferred into Matlab and an Independent Component Analysis (ICA, using EEGLAB routines)^46^ was performed to remove ocular, muscular, and electrocardiographic artefacts. Only ICA components with specific activity patterns for ocular, muscular and electrocardiographic artefacts were removed^47^. On average 7.9 ± 0.7 components were removed. In a last step, data were retransferred into NetStation to remove residual slow ocular artefacts (using Ocular Artefact Removal tool of NetStation). Further analyses were performed in Matlab. In addition to the above described pre-processing, a semiautomatic visual artefact rejection was performed based on power values in two frequency bands (0.75–4.5 Hz and 20–30 Hz). To do so the signal was down sampled to 128 Hz, and all 4-s epochs and channels containing artefacts were removed^48^. On average 23.5 ± 1.9 epochs were removed in the evening and 24.1 ± 1.9 epochs in the morning, leading to ~2.7 min of artefact free EEG recording for further analysis (evening: 2.7 ± 0.2 min; morning 2.7 ± 0.1 min). For each subject, only artefact-free channels in the morning and evening were included. For topographical analysis of theta power, the remaining data of each subject were re-referenced to an average value across all 109 channels above the ears that were not previously excluded. Theta power was calculated for each channel between 6 and 8 Hz (FFT routine, Hamming window, 4 s epochs, resolution of 0.25 Hz).

### Detection of theta events

Theta event detection was based on the average referenced EEG signal which was passband filtered in the theta frequency range (Chebyshev Type 2 Filter: pass-band 5 and 9 Hz, stopband: below 4 and above 12 Hz). An automated detection algorithm adapted from Massimini *et al*.^15^ was designed: In a first step, the signal was averaged within 4 large non-overlapping areas of the scalp (for illustration of the four areas see Fig. 1b, in ref. 15). Next, two times the standard deviation of absolute amplitudes were computed for each area and averaged across the four areas. This value was considered as detection threshold. Finally, the event detection algorithm was applied to each channel separately using the following criteria: (1) time between negative zero crossing and a subsequent positive zero crossing within 0.06–0.083 s (corresponding to 6–8 Hz), (2): negative-to-positive peak-to-peak amplitude >2 times detection threshold, (3): negative peak >detection threshold (an example of the detection algorithm is shown in Fig. 1c). In a next step, for each theta event the cluster size (i.e. number of channels involved in the theta event) was calculated. We assumed that theta waves travel across the cortex comparable to slow waves^15^. It is estimated that slow waves sweep across the entire cortex in approximately 100 ms^15^. Therefore, cluster sizes were calculated considering 5 different detection windows (20 ms–100 ms, in 20 ms bins): for each detected theta event the number of neighbouring channels involved in the given detection window was counted. For each event we assessed the following parameter: the number of channels involved in the maximal cluster size, the channel location and the respective amplitude (average across the channels within cluster).

### Alignment of performance and EEG data

The reaction times to each deviant tone were considered as behavioural variables to quantify performance. All mouse clicks occurring more than 700 ms after the presentation of the deviant tone were defined as missed clicks. To investigate the relationship between the occurrence of theta events and reaction times, a time window was defined starting 900 ms prior to the stimulus time point (deviant tone) and ending 100 ms after the stimulus time point. Therefore, only deviant tones with at least one standard tone between two deviant tones were considered (i.e. all consecutive deviant tones were excluded). Then the automated theta event detection algorithm and the calculation of cluster sizes (as described above) were repeated for this time frame. This time window of 1000 ms was selected to ensure enough data for stable theta event detection. Note, for our analysis, i.e. the direct comparison of widespread theta event occurrence prior to slow vs. fast reaction times (see below), the inclusion of the previous standard tone and the deviant tone itself within the detection window is the same for both conditions. Thus, these direct comparisons are not affected by the selected time window of 1000 ms. Nevertheless, exploratory analysis of the relationship between reaction time and widespread theta events including a time window of only 800 ms revealed similar results (data not shown).

### Statistics

Inferential statistics were computed using linear mixed effects models, because they account for covariance between related data samples in repeated measures designs^49^. The mixed effects models were fitted using restricted maximum likelihood estimation (REML) and an ante-dependent first order covariance matrix. In advance, the model fit was verified depending on model fit indices (Akaike Information Criterion and Schwarz Bayesian Criterion). F tests were used to estimate the influence of each fixed effects on the model. “*Subjects*’’ was considered as a random effect with random intercepts. For the comparison of cluster sizes between morning and evening for different detection windows a mixed effects model was performed with repeated fixed effect of “*time*’’ and “*detection window*’’. For comparison of the amplitude of different cluster sizes between morning and evening a mixed effects model with repeated fixed effect “*time*’’ and “*cluster sizes*’’ was performed. For all intra-individual comparisons, two-sided paired Student’s T-Tests were used. For exceptional comparisons with sample size of 8 or smaller, Wilcoxon signed rank test (intra-individual) or a Wilcoxon rank sum test (inter-individual) were used (indicated in the text). To control for multiple comparisons, a nonparametric cluster-based statistical testing using suprathreshold cluster analysis was performed^50–52^. In short for each topographical statistical analysis (i.e. widespread theta in the morning and evening or slow reaction times and fast reaction times) new datasets were generated by randomly relabeling the condition label from original data and paired Student’s T-Tests were performed. For each permutation the maximal size of the resulting clusters with neighbouring electrodes reaching a t-value above the predetermined critical value (CV) was counted to generate a cluster size distribution. From this cluster size distribution the 95th percentile was determined as critical cluster size threshold. For the true comparison only electrodes reaching a t-value beyond the CV and located within a cluster ≥ the critical cluster size threshold were considered as significant (n = 18, CV = 2.1, number of permutations 5000; n = 12, CV = 2.2, number of permutations 5000). Significance was set at the 5% level. All analyses were performed with the software package MATLAB (Math Works, Version 14a) or SPSS (Version 22.0, SPSS Inc. Chicago, US). Data are presented as mean ± SEM.

### Data availability

The ethical approval granted to the authors by the IRB does not allow the publication of the raw data online. If readers would like to re-analyze the data set (for different purposes), additional ethical approval (on an individual user and purpose basis) will be required. The authors would be happy to support additional ethical approval applications from researchers for access to this data set.

## Electronic supplementary material

Supplementary Information
