# Supplementary material for: Theta waves in children’s waking electroencephalogram resemble local aspects of sleep during wakefulness
Source: Sci Rep. 2017 Sep 11;7:11187. doi: 10.1038/s41598-017-11577-3 (PMC5593855; doi:10.1038/s41598-017-11577-3)
Supplement: Supplementary file 1 — Supplementary Information [file 41598_2017_11577_MOESM1_ESM.pdf]

SUPPLEMENTARY INFORMATION

Theta waves in children's waking electroencephalogram resemble local aspects of sleep during wakefulness

Sara Fattinger [1, 2, 3], Salome Kurth [2, 3, 4], Maya Ringli [1, 2, 3], Oskar G. Jenni [1, 2, 3], Reto Huber[1, 3, 5]

1) Child Development Center, University Children's Hospital Zurich, Switzerland

2) Children's Research Center, University Children's Hospital Zurich, Switzerland

3) Neuroscience Center Zurich, Zurich, Switzerland

4) Pulmonary Clinic, Division of Pulmonology, University Hospital Zurich, Switzerland

5) Department of Child and Adolescent Psychiatry and Psychotherapy, Psychiatric Hospital, University of Zurich, Switzerland

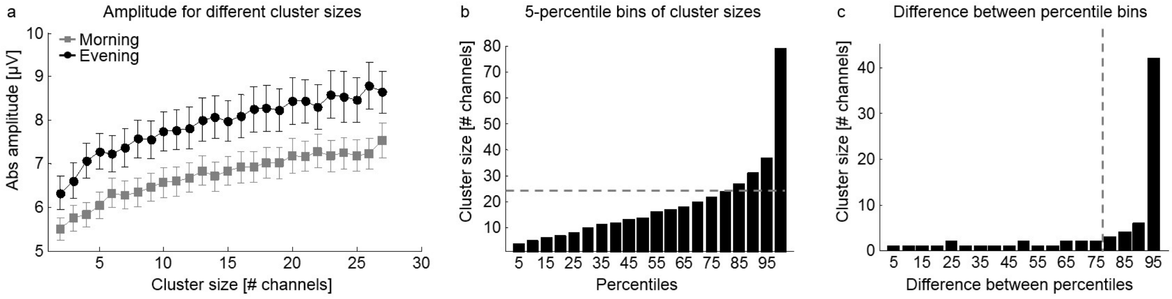

Figure S1

Definition of widespread theta events based on a detection window of 100ms.

(a) Relationship between amplitude and cluster size in the morning and evening (mean ± SEM of the amplitude for each cluster size are presented for morning and evening. For each subject, mean amplitude was calculated when at least 5 theta events for the given cluster size were detected). Larger amplitudes were associated with increased cluster sizes (linear mixed effect model $F_{cluster\ size}$=43.2, p<0.001, n=12). For any cluster size a larger amplitude was detected in the evening compared to morning (linear mixed effect model: F_{time}=71.52, p<0.001, n=12). (b) 5-percentile bins of cluster sizes (morning and evening pooled). The 80th percentile corresponds to a cluster size of 24 channels (grey dotted line). (c) Difference in the number of channels involved in a cluster size between each 5-percentile bin from Figure b. Numbers on the x-axis indicate the lower bin (i.e., 5 corresponds to the difference between the 5th and the 10th bin). Because the number of channels involved in the cluster size are increasing from the 80th percentile, the 80th percentile (corresponding to a cluster size of 24 channels, see b) was used for cluster size cutoff definition for widespread theta events.

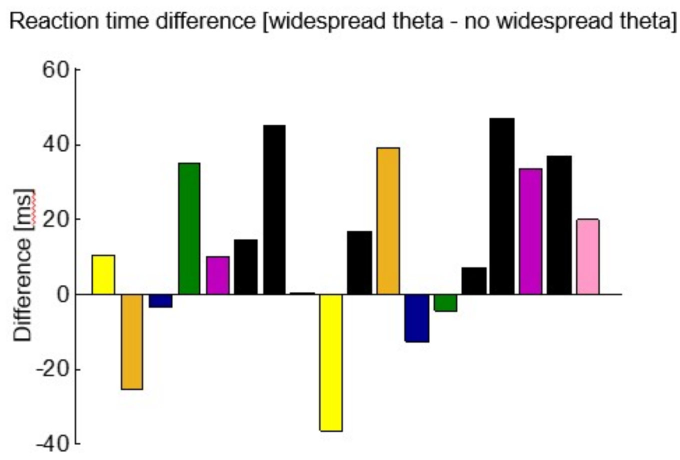

Figure S2

Intra-individual comparison of reaction time.

Difference in reaction time associated with widespread theta (i.e. at least one widespread theta event) and reaction time without widespread theta for each intra-individual comparison. Since data of the morning and evening were pooled some subjects were considered twice (same colour=same subject, the first 8 comparisons correspond to the morning session). Note, the slower reaction times for stimuli associated with widespread theta seems not to be driven by the subjects which were included twice.

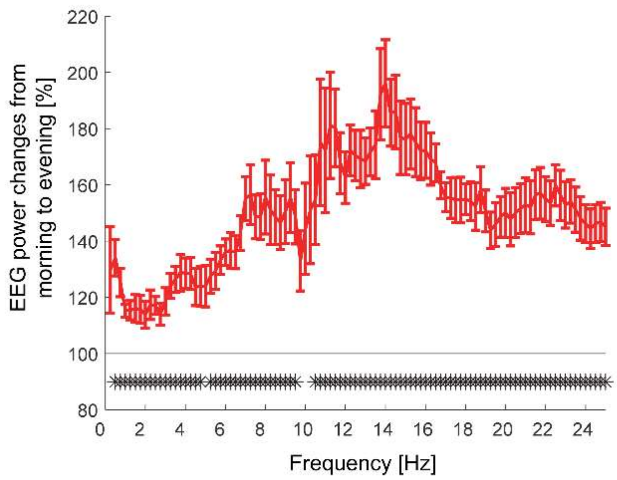

Figure S3

EEG power changes from morning to evening.

Mean spectral power (mean over all electrodes, 0.25-25Hz, divided in 0.25Hz bins) of the evening expressed as percentage of the morning (mean ± SEM; 100 = power spectrum in the morning, black stars represent frequencies with $p < 0.05$, paired Student's T-Test; n=12). Note, a similar increase of spectral power was observed in adults after sleep deprivation under constant routine conditions (i.e. disentangle wake and circadian dependent effects, Aeschbach et al.[4]). The authors found a wake-dependent increase of power density between 0.75-9Hz and 12.25-25 Hz, whereas no circadian modulation was found for the 12.25-25Hz band. Thus, compared to adults, in prepubertal children increasing sleep pressure in the evening might uncover the circadian modulation on the sigma and beta band.
